# Supplementary material for: Hemangiosarcoma Cells Promote Conserved Host-derived Hematopoietic Expansion
Source: Cancer Res Commun. 2024 Jun 11;4(6):1467–80. doi: 10.1158/2767-9764.CRC-23-0441 (PMC11166094; doi:10.1158/2767-9764.CRC-23-0441)
Supplement: Supplementary Figure S1 [file crc-23-0441-s01.pdf]

Supplementary Figure S1

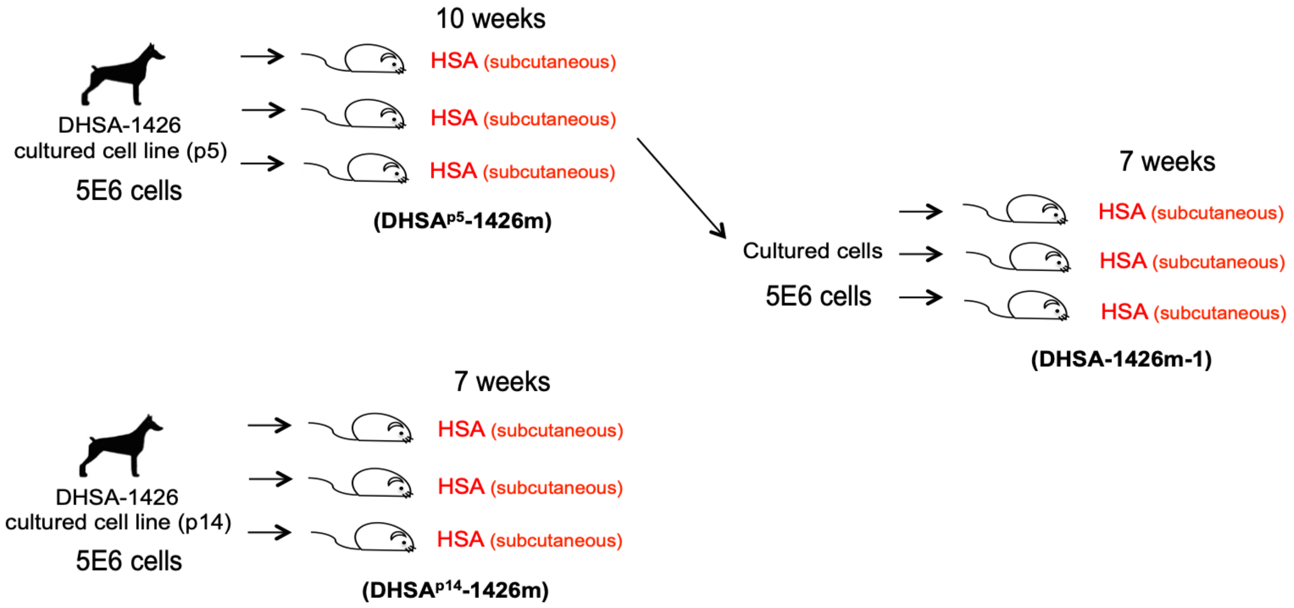

Supplementary Figure S1. Serial transplantation of canine hemangiosarcoma cells in immunodeficient mice. Xenograft tumors were reproducible by inoculation of passage 5 (p5) and p14, DHSA-1426 cells. DHSA-1426 cells from p5 were serially passaged into new recipient mice and tumor development was accelerated from 10 weeks in the initial xenografts to seven weeks in the first passage xenografts. The additional time in culture (from p5 to p14) also accelerated development of xenografts from 10 to seven weeks.
