# Supplementary material for: Hemangiosarcoma Cells Promote Conserved Host-derived Hematopoietic Expansion
Source: Cancer Res Commun. 2024 Jun 11;4(6):1467–80. doi: 10.1158/2767-9764.CRC-23-0441 (PMC11166094; doi:10.1158/2767-9764.CRC-23-0441)
Supplement: Supplementary Figure S2 [file crc-23-0441-s02.pdf]

Supplementary Figure S2

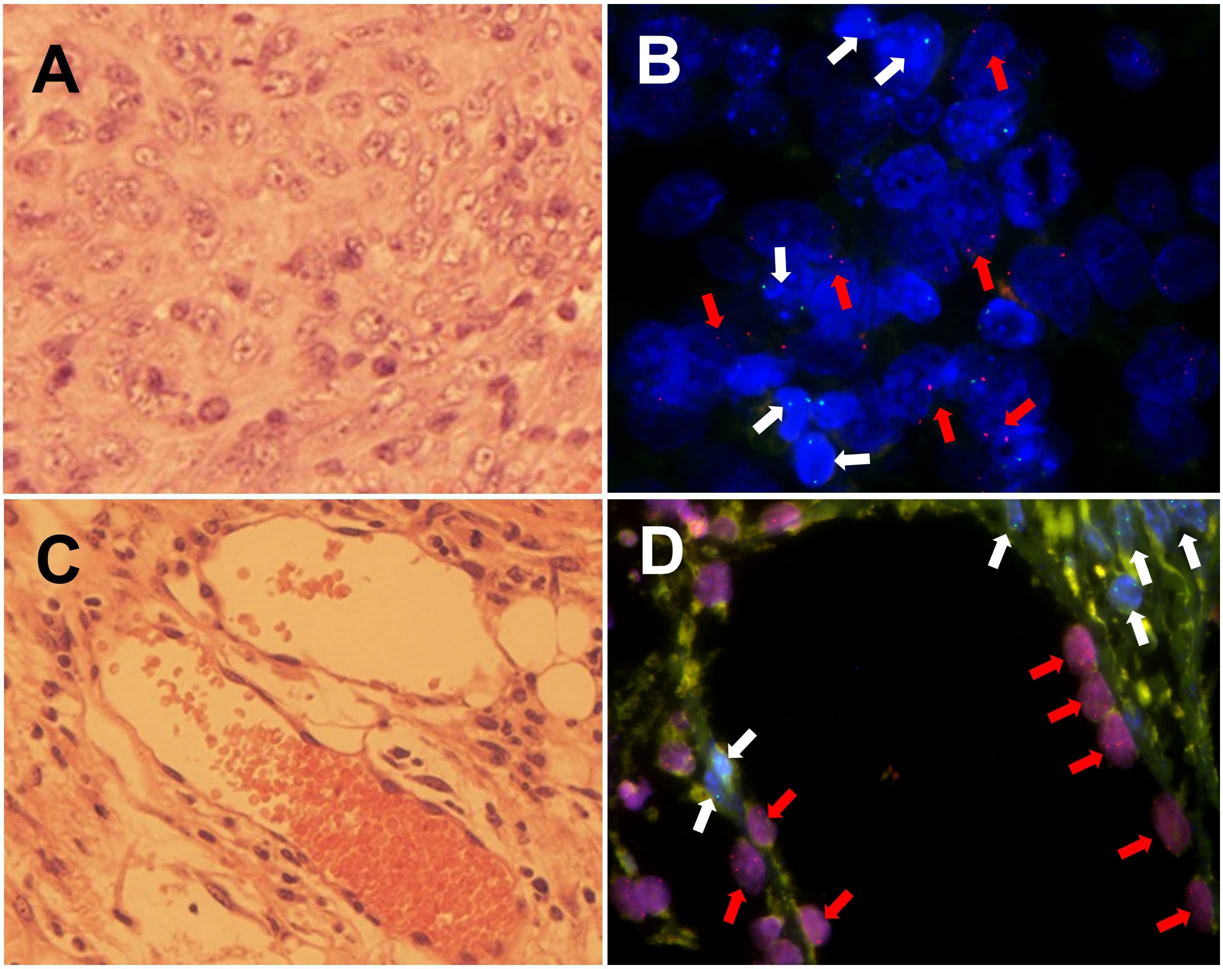

Supplementary Figure S2. Organization of tumor and stromal cells in mouse xenografts of canine hemangiosarcoma. Representative microphotographs show that hemangiosarcoma tissues formed in xenografts contain two distinct areas: the tumor-propagating area (**A** and **B**) and microvascular channels (**C** and **D**) in adjacent tumor. (A and C) H&E stained histological images were acquired using a bright-field microscope. (B and D) Fluorescence *in situ* hybridization images were obtained using canine-specific (*CXCL8*, red) and mouse-specific (X chromosome, green) probes in canine hemangiosarcoma xenografts, transplanted into receptive immunodeficient female mouse hosts. Red and white arrows point to representative xenograft canine tumor cells and mouse stromal cells, respectively, to aid in identification.
