# Supplementary material for: Hemangiosarcoma Cells Promote Conserved Host-derived Hematopoietic Expansion
Source: Cancer Res Commun. 2024 Jun 11;4(6):1467–80. doi: 10.1158/2767-9764.CRC-23-0441 (PMC11166094; doi:10.1158/2767-9764.CRC-23-0441)
Supplement: Supplementary Figure S3 [file crc-23-0441-s03.pdf]

Supplementary Figure S3

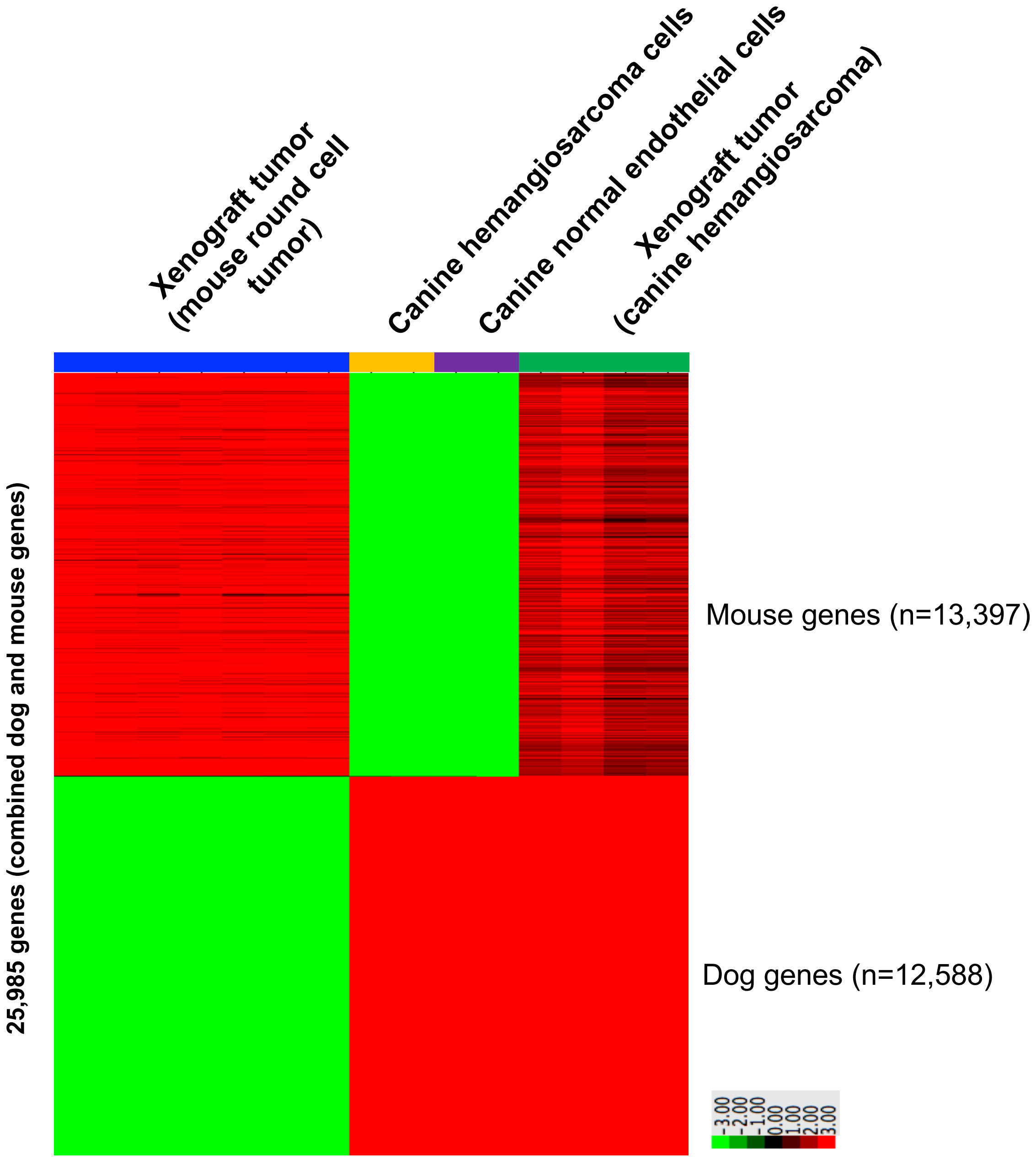

Supplementary Figure S3. Identification of dog tumor and mouse stromal signatures from hemangiosarcoma xenografts. We identified donor (dog) and host (mouse) genes in mouse round cell tumors (N = 7; blue column bar) and hemangiosarcoma xenografts (N = 4; green column bar) using RNA-seq and our dog/mouse hybrid genome bioinformatics pipeline. Sequencing reads to canine and murine genes within xenograft tumors were mapped to dog (canFam3) and mouse reference genome (mm10) using a HISAT2. Species-specific gene counts were calculated as described. Canine hemangiosarcoma cells (N = 2; yellow column bar) and normal endothelial cells (CnAOEC; N = 2; purple column bar) cultured in *in vitro* were used as controls. A heat map shows up-regulated (red) and down-regulated (green) genes between genes by unsupervised hierarchical clustering (average linkage; mean- centered; log2 transformed).
