# Supplementary material for: Hemangiosarcoma Cells Promote Conserved Host-derived Hematopoietic Expansion
Source: Cancer Res Commun. 2024 Jun 11;4(6):1467–80. doi: 10.1158/2767-9764.CRC-23-0441 (PMC11166094; doi:10.1158/2767-9764.CRC-23-0441)
Supplement: Supplementary Figure S4 [file crc-23-0441-s04.pdf]

Supplementary Figure S4

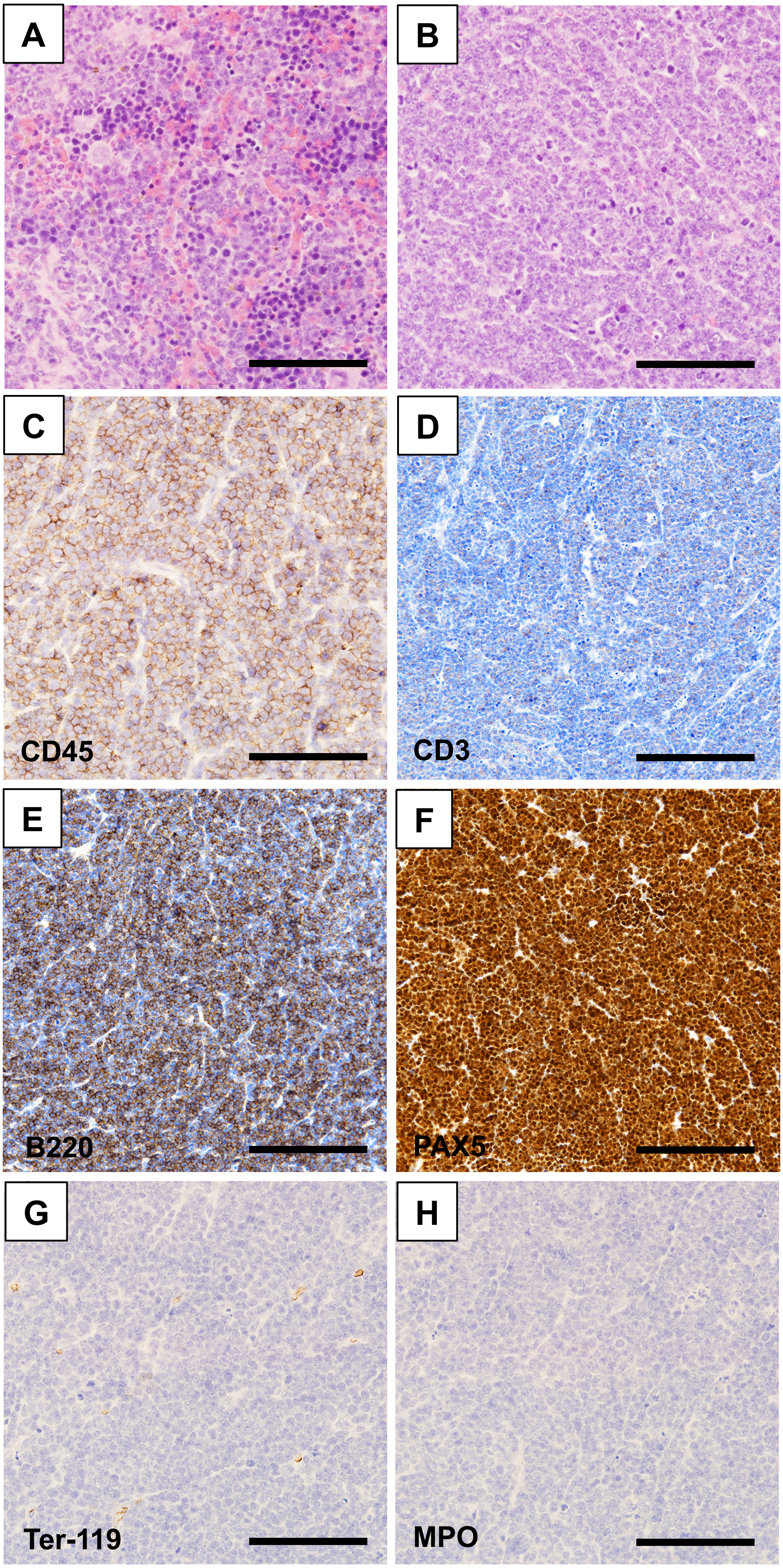

Supplementary Figure S4. Round cell tumor developed from xenotransplantation of canine hemangiosarcoma in immunodeficient mice. (**A** and **B**) Representative photomicrographs display histopathology of tumors from BNX mice transplanted with DHSA-1426 hemangiosarcoma cell line. H&E stain; splenic tumor (**A**) and tumor in lymph node (**B**). Immunohistochemistry was done in lymph node tumors with anti-CD45 (**C**), anti-CD3 (**D**), anti-B220 (**E**), anti-PAX5 (**F**), anti-Ter-119 (**G**), and anti-MPO (**H**) antibodies. Horseradish peroxidase conjugates were used. Counterstain = hematoxylin. Bar = 50 µm.
