# Supplementary material for: Hemangiosarcoma Cells Promote Conserved Host-derived Hematopoietic Expansion
Source: Cancer Res Commun. 2024 Jun 11;4(6):1467–80. doi: 10.1158/2767-9764.CRC-23-0441 (PMC11166094; doi:10.1158/2767-9764.CRC-23-0441)
Supplement: Supplementary Figure S5 [file crc-23-0441-s05.pdf]

Supplementary Figure S5

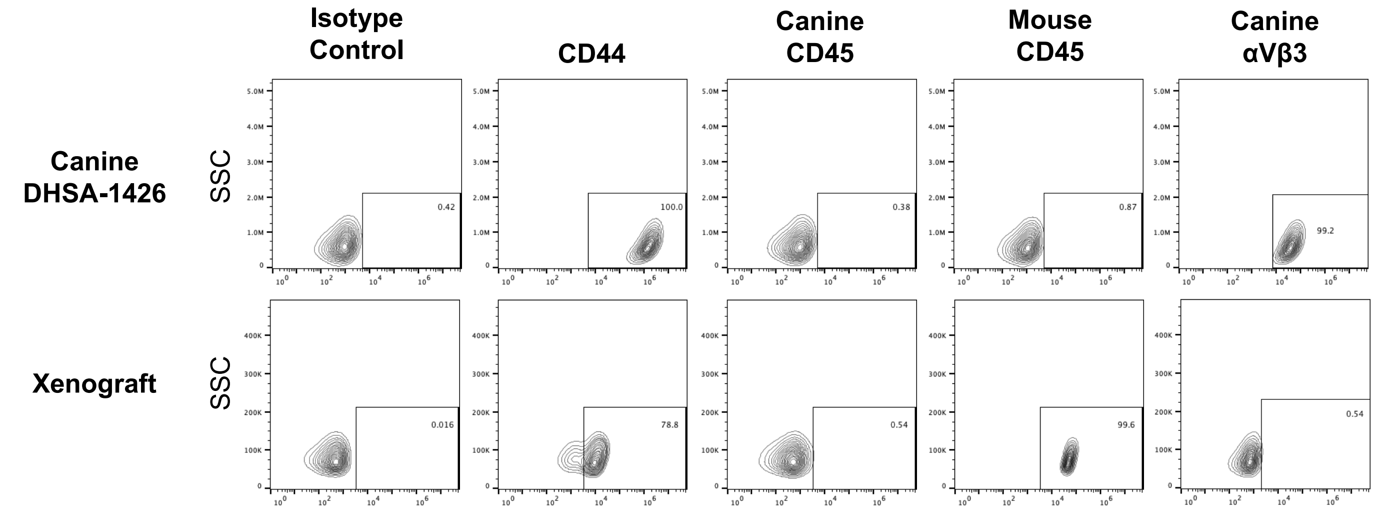

Summary of staining

	CD44	Canine CD45	Mouse CD45	Canine αVβ3
**DHSA-1426 cells**	High	Low	Low	High
**Xenograft tumor cells**	High	Low	High	Low

Supplementary Figure S5. Flow cytometric analysis of mouse round cell tumors from xenograft of canine hemangiosarcoma. Contour plots show cell population stained with anti-canine CD45, anti-mouse CD45, and anti-human αVβ3-integrin antibodies in canine DHSA-1426 cells and mouse round cell tumors. Anti-CD44 antibody (dog- and mouse-cross-reactive) was used as staining positive control.
