# Supplementary material for: Hemangiosarcoma Cells Promote Conserved Host-derived Hematopoietic Expansion
Source: Cancer Res Commun. 2024 Jun 11;4(6):1467–80. doi: 10.1158/2767-9764.CRC-23-0441 (PMC11166094; doi:10.1158/2767-9764.CRC-23-0441)
Supplement: Supplementary Figure S6 [file crc-23-0441-s06.pdf]

Supplementary Figure S6

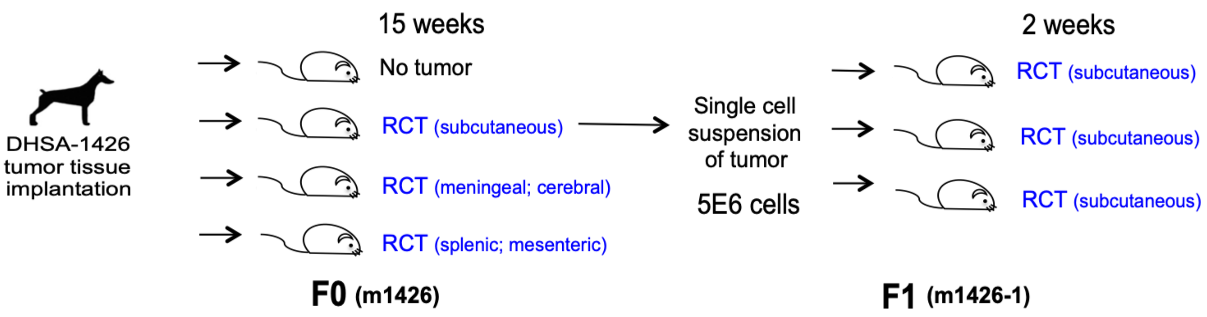

Supplementary Figure S6. Schematic illustration of incidence of mouse round cell tumor and hemangiosarcoma in xenotransplantation of canine hemangiosarcoma in immunodeficient mice. Splenic tumor fragment obtained from a dog with hemangiosarcoma was surgically implanted into the flank of BNX mice. Three of four mice developed round cell tumors at 15 weeks. After the tumors were harvested, 5×10^6 tumor cells in single cell suspension were implanted into three mice, and all of the mice developed round cell tumors two weeks later.
