# Supplementary material for: Hemangiosarcoma Cells Promote Conserved Host-derived Hematopoietic Expansion
Source: Cancer Res Commun. 2024 Jun 11;4(6):1467–80. doi: 10.1158/2767-9764.CRC-23-0441 (PMC11166094; doi:10.1158/2767-9764.CRC-23-0441)
Supplement: Supplementary Figure S7 [file crc-23-0441-s07.pdf]

Supplementary Figure S7

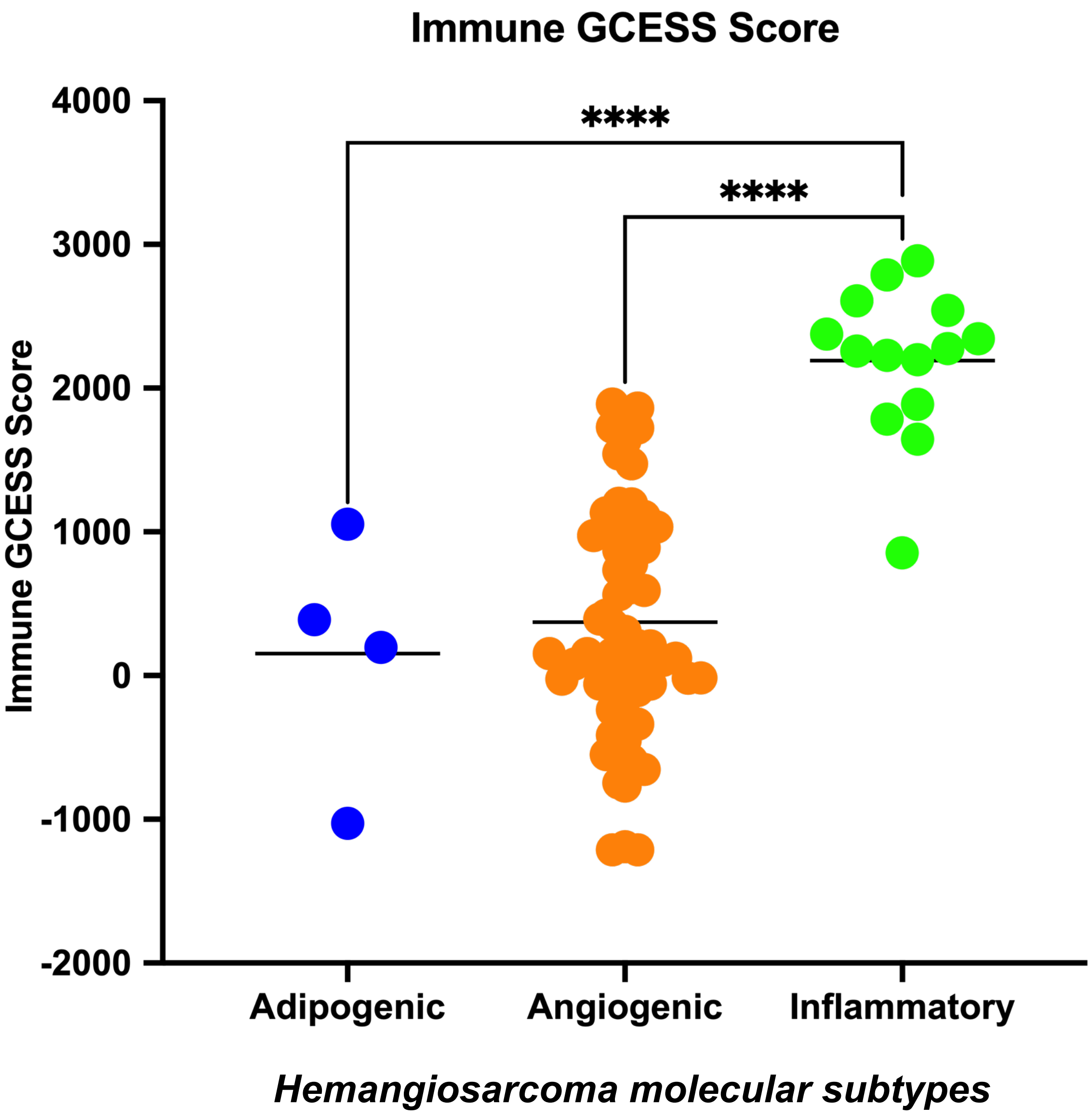

Supplementary Figure S7. Immune GCESS scores in RNA-seq data of canine hemangiosarcoma tissues. (**A**) A total of 76 canine hemangiosarcomas were analyzed for immune GCESS scores (adipogenic, N=4; angiogenic, N=58; inflammatory, N=14). Three molecular subtypes of canine hemangiosarcomas were identified as previously described [Ref 4,8]. One-way ANOVA test; ****, P<.0.0001
