# Supplementary material for: Hemangiosarcoma Cells Promote Conserved Host-derived Hematopoietic Expansion
Source: Cancer Res Commun. 2024 Jun 11;4(6):1467–80. doi: 10.1158/2767-9764.CRC-23-0441 (PMC11166094; doi:10.1158/2767-9764.CRC-23-0441)
Supplement: Supplementary Figure S8 [file crc-23-0441-s08.pdf]

Supplementary Figure S8

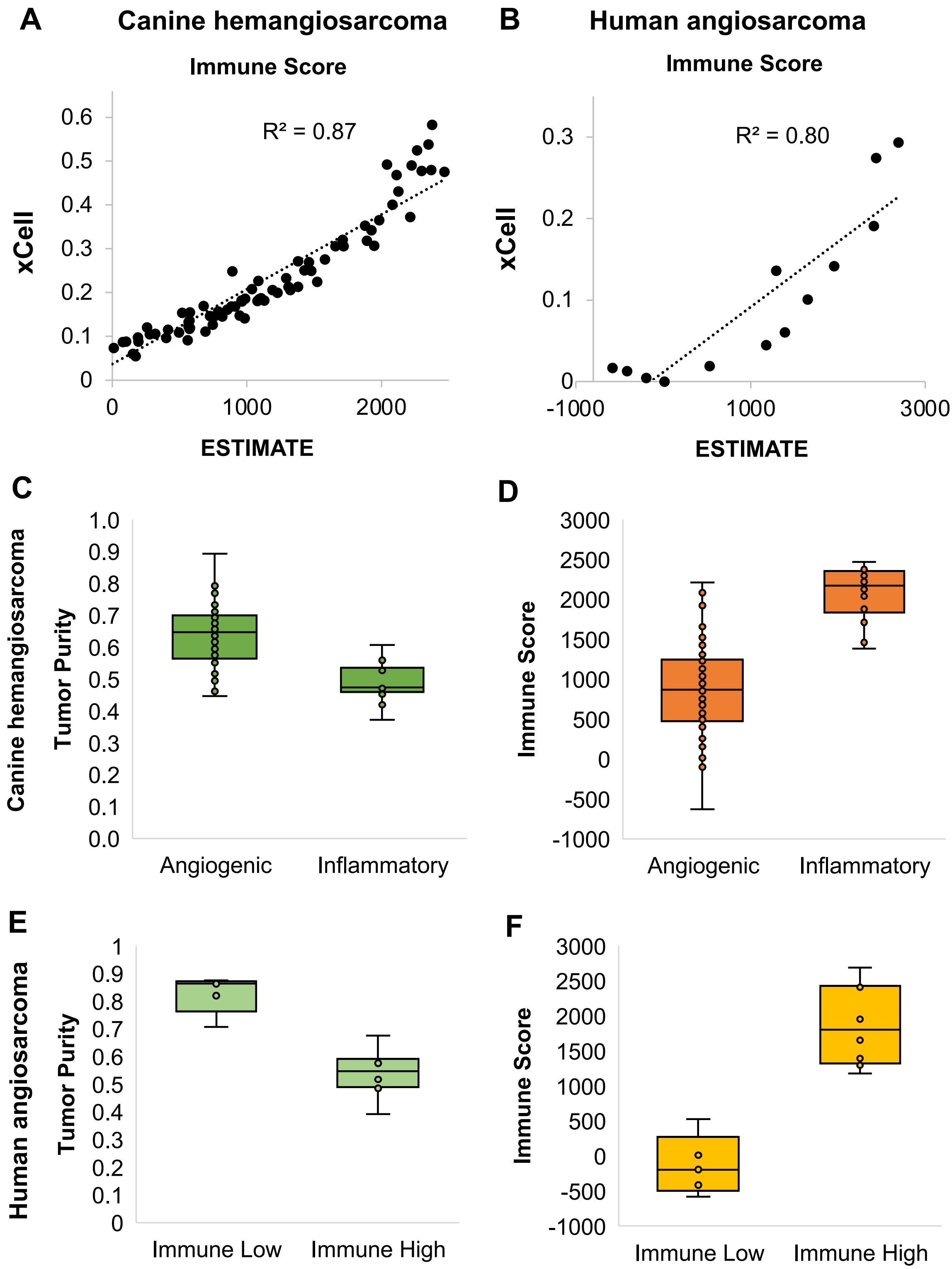

Supplementary Figure S8. Transcriptional immune and tumor purity score in canine hemangiosarcoma and human angiosarcoma. (**A** and **B**) Scatter plots display correlation between immune scores predicted by ESTIMATE (x-axis) and *xCell* (y-axis) tools in canine hemangiosarcoma (**A**) and human angiosarcoma (**B**). Coefficient of determination (R2) was calculated by linear regression. (**C** - **F**) Box and Whisker plots show tumor purity and immune scores between canine angiogenic and inflammatory hemangiosarcoma (**C** and **D**) and between human angiosarcoma with low and high immune signature (**E** and **F**).
