# Supplementary material for: Hemangiosarcoma Cells Promote Conserved Host-derived Hematopoietic Expansion
Source: Cancer Res Commun. 2024 Jun 11;4(6):1467–80. doi: 10.1158/2767-9764.CRC-23-0441 (PMC11166094; doi:10.1158/2767-9764.CRC-23-0441)
Supplement: Supplementary Figure S9 [file crc-23-0441-s09.pdf]

Supplementary Figure S9

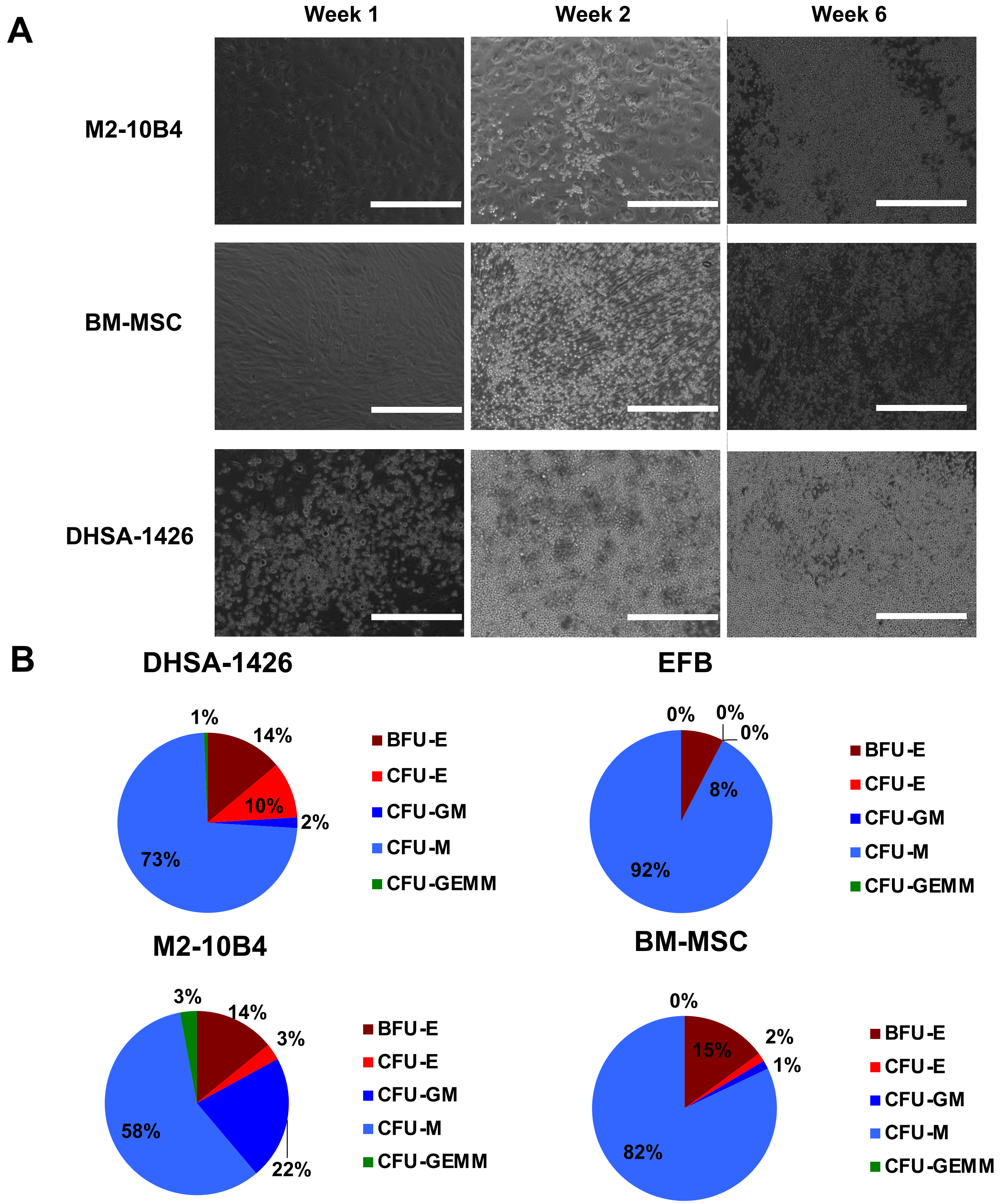

Supplementary Figure S9. Long-term culture initiating cell and hematopoietic colony-forming unit assays on CD34+ cells and canine hemangiosarcoma cells. (A) Representative photomicrographs display proliferation of CD34+ hUCB cells co-cultured with M2-10B4, BM-MSC, and DHSA-1426 cells at week 1, 2 and 6. Bar = 400 µm. (**B**) Pie charts present colony-forming units differentiated from CD34+ hUCB cells by co-culture with different feeder cells, DHSA-1426, EFB, M2-10B4, and BM-MSC.
