# Supplementary material for: Hemangiosarcoma Cells Promote Conserved Host-derived Hematopoietic Expansion
Source: Cancer Res Commun. 2024 Jun 11;4(6):1467–80. doi: 10.1158/2767-9764.CRC-23-0441 (PMC11166094; doi:10.1158/2767-9764.CRC-23-0441)
Supplement: Supplementary Figure S10 [file crc-23-0441-s10.pdf]

Supplementary Figure S10

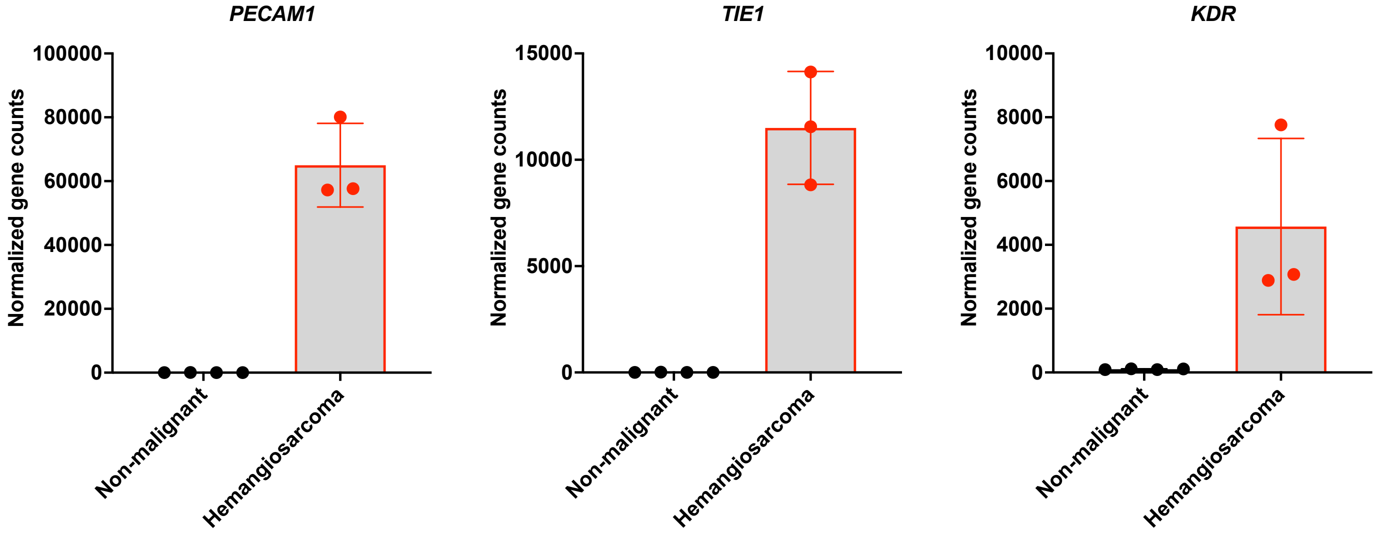

Supplementary Figure S10. Expression of representative marker genes for endothelial progenitors in DHSA-1426 hemangiosarcoma cells. Bar graphs display normalized count values for *PECAM1*, *TIE1*, and *KDR* genes in RNA-seq data generated from DHSA-1426 cells (n=3) and non-malignant endothelial cells (n=4).
